# Editorial: Pathogenesis, diagnostics, treatments of *Mycobacterium tuberculosis* and its co-infection with HIV or SARS-CoV-2

**DOI:** 10.3389/fcimb.2024.1359356

**Published:** 2024-01-18

**Authors:** Divakar Sharma, Amit Singh

**Affiliations:** ^1^ Department of Microbiology, Lady Hardinge Medical College, Delhi University, Delhi, India; ^2^ Department of Microbiology, Central University of Punjab, Bathinda, Punjab, India

**Keywords:** antimicrobial resistance, tuberculosis, co-infection, HIV, SARS-CoV-2

In this Research Topic of Frontiers in Cellular and Infection Microbiology and Frontiers in Immunology, we edited a collection of eight original research articles and one case report within the theme “*Pathogenesis, Diagnostics, Treatments of Mycobacterium tuberculosis and It’s Co-Infection with HIV or SARS-CoV-2*.” The major goal of this Research Topic was to gather the updated view of the modern approaches used for the early diagnosis, treatment and pathogenesis mechanisms of *M. tuberculosis* and its co-infection with HIV or SARS-CoV-2, which ultimately delivered the updated view on technologies and their applications for the management of these co-infections. *Mycobacterium tuberculosis* is the major cause of tuberculosis (TB) disease across the globe. One-fourth of the world’s population is infected with TB asymptomatically. Longer regimen of anti-TB drugs (leading to poor adherence and treatment), interrupted anti-TB drugs treatment (incomplete anti-TB treatment), and ineffectiveness of the anti-TB drugs due to the re-emergence of latent TB infections are major issues that hindrance to achieving the end of the global TB epidemic by 2035 as WHO plans. The emergence of drug-resistant *Mycobacterium tuberculosis* and co-infections with HIV as well as SARS-CoV-2 poses a serious threat to global health agencies. It was reported that the TB cases in India and other endemic countries are 2-3 times higher than in the last few years. Bacteria have acquired different mechanisms and became multidrug-resistant by various mechanisms like alternation in the target site, over expression of efflux pumps, inactivation of drugs by enzymes and biofilms (Singh et al., 2015). These mechanisms adopted by bacteria and longer anti-tuberculosis treatment regimens are the greatest threat in TB control programs especially in malnourished, immune-compromised, *Mycobacterium tuberculosis* co-infection with HIV and SARS-CoV-2 in developing countries. The incidence of infectious diseases like Tuberculosis & its drug resistant forms, and co-infection with HIV & SARS-CoV-2 are high and often cause critical illness, which seriously threatens human life and health. In this editorial, we have focus on this thematic issue and discussed the edited papers, which help to culminate this serious problem.

Early diagnosis of mycobacterium is crucial for the management of clinical tuberculosis. Li et al. evaluated and validated the diagnostic performance like sensitivity, specificity, and accuracy of the nucleotide matrix-assisted laser desorption time-of-flight mass spectrometry (MALDI-TOF-MS) for identification of mycobacterium species. They analyzed 108 samples, and found the 96.91% sensitivity, and 100% specificity of the nucleotide MALDI-TOF-MS in mycobacterial identification and the lower limit of detection was 50 bacteria/mL. For validation of *Mycobacterium tuberculosis* diagnosis they have compared the sensitivity and specificity of MALDI-TOF-MS (72.7%, 100%), to the Gene-Xpert (63.6%, 100%), culture (54.5%, 100%), and AFS (27.3%, 100%). Further they have suggested that the optimized nucleotide MALDI-TOF-MS has higher sensitivity, and specificity at lower limit of detection in mycobacterial identification, and would considered as a potential assay for identification.

Tang et al. evaluated the Interferon-inducible protein 10 (IP-10) m-RNA release assay for the diagnosis of TB in patients with HIV co-infection and efficacy compared to the Interferon gamma release assays (IGRAs) test (QFT-GIT). The IP-10 mRNA release assay was significantly higher sensitive than that of QFT-GIT test, because IP-10 mRNA release assay is less dependent on CD4+ T cells count (a well stated attribute of HIV-infected patients) than QFT-GIT test. Therefore, this study suggested that *M.tuberculosis* specific IP-10 mRNA is considered as a better biomarker for diagnosis of TB in patients with HIV co-infection.

Xu et al. compared the diagnostic performance of laboratory assays like GeneXpert, culture, and T-SPOT.TB (T-SPOT) on the ultrasound-guided core needle biopsy samples for diagnosis of extra-pulmonary tuberculosis (EPTB) in HIV-positive and HIV-negative patients. For ultrasound-guided core needle biopsy samples, GeneXpert (Xpert) showed higher sensitivity and specificity than culture and T-SPOT.TB (T-SPOT), which suggested the superior performance of GeneXpert as compared to other method in diagnosing EPTB across HIV status and sample types.

Hu et al. reported the diagnostic efficacy of the QuantiFERON-TB Gold In-Tube (QFT-GIT) test for the preoperative differential diagnosis of spinal tuberculosis. They have analyzed the patients with spinal tuberculosis by the QFT-GIT test and found the sensitivity and the specificity were 92.16% and 67.14% respectively. They have increased the accuracy of diagnostic QFT-GIT test from 77.42% to 80.65% when a new threshold (1.58 IU/mL) assigned for the study cohort and suggested that the intensity of the QFT-GIT test findings in spinal tuberculosis may be related to the duration of a patient’s disease.

In a study, Singh et al. accessed the distribution of *M.tuberculosis* genotypes among HIV-positive and HIV-negative patients suspected to tuberculosis at the National Institute of Tuberculosis and Respiratory Diseases, New Delhi, a tertiary care dedicated TB hospital. They screened the 503 subjects for pulmonary and extra-pulmonary tuberculosis, and found 276 *M. tuberculosis* culture positive. Genotyping data revealed that most prevalent lineage was Central Asian Strain (CAS) genotype followed by Beijing genotype among HIV-positive and HIV-negative patients. Through in this study they reported that predominance of Beijing genotype was almost double (22.5%) in HIV-positive as compare to the HIV-negative (10.9%), which suggest the high transmissibility of these genotypes in the community.

In another study, de Sa et al. investigated the relationship between from single nucleotide polymorphisms (SNPs) of the NLRP3, CARD8, AIM2, CASP-1, IFI16, and IL-1b inflammasome genes, as well as the profiles of secreted pro-inflammatory cytokines (e.g., IL-1b, IL-18, IL-33, and IL-6) with the TB clinical profiles, TB-HIV co-infection, and IRIS onset. TT genotype in NLRP3 rs4612666 and C-C-T-G-C NLRP3 haplotype polymorphisms were associated with the increased risk for EPTB, however increased levels of IL-6 or IL-33 and IL-18 or IL-33 were found in TB patients both without and with HIV carrying the minor frequency allele NLRP3 polymorphisms. Therefore, this study depicted the association of genetic polymorphisms (Single or multiple) of crucial genes of innate immunity as well as pro-inflammatory cytokines in the clinical outcomes related to co-infections like TB-HIV.

Weng et al. presented a case report on disseminated tuberculosis in a child during the COVID-19 pandemic and hypothesized that COVID-19 infection created immunosuppressive effect with possible implications for tuberculosis dissemination. Further they suggested that more significant research to be needed for better diagnosis and treatment options for the co-infection of *M. tuberculosis* and SARS-CoV-2.

Huang et al. elucidated the common processes and pathways between COVID-19 and TB pathogenesis through bioinformatics and system biology approaches. They used RNA-seq datasets (GSE196822 and GSE126614) and identified differentially expressed genes (DEGs) IFI44L, ISG15, MX1, IFI44, OASL, RSAD2, GBP1, OAS1, IFI6, and HERC5. Further they identified some potential drugs (suloctidil, prenylamine, acetohexamide, terfenadine, prochlorperazine, 3′-azido-3′-deoxythymidine, chlorophyllin, etoposide, clioquinol, and propofol), which to be proposed as alternative treatment strategy for TB and COVID-19, after their validation by future research.

In conclusion, this Research Topic includes several excellent studies summarizing the latest advances in the diagnosis and therapeutics of pulmonary tuberculosis, extra-pulmonary tuberculosis co-infection with HIV and SARS-CoV-2. This Research Topic will promote the integration of the advanced approaches (genomics, transcriptomics, proteomics and bioinformatics) for the development of superior diagnostics and therapeutics, in future, which would be used in the management of tuberculosis, and co-infection with HIV and SARS-CoV-2 (Sharma et al, 2020). Finally, to fight with these pandemics (TB, COVID-19 and HIV) developing new diagnostic approaches should consider the economic component of low and middle-income countries for universal equality and one health world.

## Author contributions

DS: Conceptualization, Supervision, Writing – original draft, Writing – review & editing. AS: Writing – review & editing.
